# Three-dimensional atrous inception module for crowd behavior classification

**DOI:** 10.1038/s41598-024-65003-6

**Published:** 2024-06-22

**Authors:** Jong-Hyeok Choi, Jeong-Hun Kim, Aziz Nasridinov, Yoo-Sung Kim

**Affiliations:** 1 Bigdata Research Institute, Chungbuk National University, Cheongju, 28644 South Korea; 2 Research Institute, AICON Company Co., Ltd, Seoul, 06774 South Korea; 3 Department of Computer Science, Chungbuk National University, Cheongju, 28644 South Korea; 4 Department of Artificial Intelligence, Inha University, Incheon, 22212 South Korea

**Keywords:** Computer science, Information technology

## Abstract

Recent advances in deep learning have led to a surge in computer vision research, including the recognition and classification of human behavior in video data. However, most studies have focused on recognizing individual behaviors, whereas recognizing crowd behavior remains a complex problem because of the large number of interactions and similar behaviors among individuals or crowds in video surveillance systems. To solve this problem, we propose a *three-dimensional atrous inception module* (*3D-AIM*) network, which is a crowd behavior classification model that uses atrous convolution to explore interactions between individuals or crowds. The *3D-AIM* network is a 3D convolutional neural network that can use receptive fields of various sizes to effectively identify specific features that determine crowd behavior. To further improve the accuracy of the* 3D-AIM* network, we introduced a new loss function called the *separation loss* function. This loss function focuses the *3D-AIM* network more on the features that distinguish one type of crowd behavior from another, thereby enabling a more precise classification. Finally, we demonstrate that the proposed model outperforms existing human behavior classification models in terms of accurately classifying crowd behaviors. These results suggest that the *3D-AIM* network with a *separation loss* function can be valuable for understanding complex crowd behavior in video surveillance systems.

## Introduction

In the era of computer vision, various studies have been conducted over a long period to enable computers to understand human behavior. The advent of deep learning has enabled computers to understand human behavior more accurately^1^. In particular, deep learning-based studies have mainly focused on recognizing or classifying individual human behaviors from video datasets, such as Kinetics-400^2^, comprising a few humans, and classifying human behavior with high accuracy using convolutional neural networks (CNN)^3^. In these studies, various human behavior classification models, such as MoViNet^4^, SlowFast^5^ and X3D^6^ have been proposed and are widely used. Additionally, researchers have recently proposed new methods, such as ViViT^7^, which uses a vision transformer (ViT)^8^ to apply the transformer architecture from natural language processing to the field of computer vision.

However, applying these studies to monitor real-world situations, such as human behavior or dangerous situations, using existing video surveillance systems has several challenges. Unlike video datasets used in previous studies to classify human behavior, video datasets targeting the real world, such as CCTV, include several crowds of people, and it is challenging to predict human movement or interactions between humans and crowds^9,10^. Therefore, recognizing or classifying crowd behavior and understanding the situation in a crowd are challenging because of the similar and complex interactions between humans and crowds in the real world^11–19^. Consequently, using newly proposed video datasets and models, recent studies have focused on accurately classifying crowd behaviors or situations. The most representative dataset proposed for this is the Crowd-11 dataset^10,20^; many studies have performed crowd behavior classification using existing human behavior classification models on the crowd-11 dataset^9,21–23^. However, despite these efforts, achieving sufficient crowd behavior classification remains a challenge owing to the diverse characteristics exhibited by different crowds. The complexity and variability of crowd behavior make it difficult to achieve accurate and robust classification results. Consequently, further advancements are required to overcome these challenges and improve crowd behavior classification.

In this study, we introduced a novel three-dimensional (3D) CNN-based model for crowd behavior classification, called the *three-dimensional atrous inception module* (*3D-AIM*) network, to improve the accuracy of video surveillance systems. The proposed *3D-AIM* network employs an atrous convolution^14–16,24^ instead of a typical convolution, which was used in previous human behavior classification models. This is because a typical convolution cannot extract the interaction features between humans or crowds beyond the receptive field, leading to inaccurate classification. By contrast, atrous convolution can extract features from receptive fields of varying sizes^25^, enabling the network to consider a broader range of interactions between humans or crowds in the real world. Therefore, our *3D-AIM* network can effectively analyze the interactions between humans or crowds through atrous convolution, leading to more accurate crowd behavior classification.

Furthermore, we introduce a new *separation loss* function that enhances the precision of crowd behavior classification by allowing the *3D-AIM* network to concentrate on behavioral features that define crowd behavior classes. The *separation loss* method calculates the loss value by maximizing the difference between the target and remaining classes based on the prediction of each class. This mechanism helps the *3D-AIM* network pay more attention to the distinct characteristics of each crowd behavior class, leading to more accurate crowd behavior classification compared to conventional loss functions used for classification tasks.

This study aims to show a more accurate crowd behavior classification in video surveillance systems used in real-world environments and the possibility of deep learning-based automated video surveillance systems needed to understand crowd situations.

The remainder of this paper is organized as follows. Sect “Related work” introduces the datasets used for crowd behavior classification and explores the existing crowd behavior classification models and loss functions. Sect “*Three-dimensional atrous inception module* (*3D-AIM*)” describes the proposed *3D-AIM* network, while Sect “*Separation loss*” introduces the novel *separation loss* function. In Sect “Performance evaluation”, we present the results of a comparative experiment between the proposed and existing methods and demonstrate the superiority of the proposed approach. Finally, Sect “Conclusion” summarizes the key contributions of the study.

## Related work

In this section, we provide an overview of the Crowd-11 dataset and existing studies that have used it to classify crowd behavior. Next, we describe the loss functions used in the previous studies and their limitations.

### Crowd-11 dataset

Crowd monitoring in video surveillance systems is of considerable interest in preventing dangerous situations or excessive crowd gatherings. However, practically, utilizing existing research and data on human behavior can be challenging because many studies^3–5,7,8^ focus on videos with a few people and may not capture the diverse interaction among humans in crowds^26^. These interactions, which can be represented as crowd flow^16–19,27,28^, assume various forms based on human navigation of space, avoidance of each other, and communication with each other when necessary. For example, crowds move toward their destination while avoiding each other to avoid collision, and in this process, people moving in the same direction may form large groups. They also change their walking speed or direction depending on the distance from the people around them. These interactions generate diverse and difficult-to-unify characteristics, making it challenging to apply existing research and data to real-world situations. This is because factors, such as crowd density, anonymity, and situational cues can affect the behavior and interactions within a crowd. Therefore, more robust and comprehensive datasets and methodologies that can effectively capture and classify crowd behavior in complex and dynamic environments are required.

**Figure 1:**
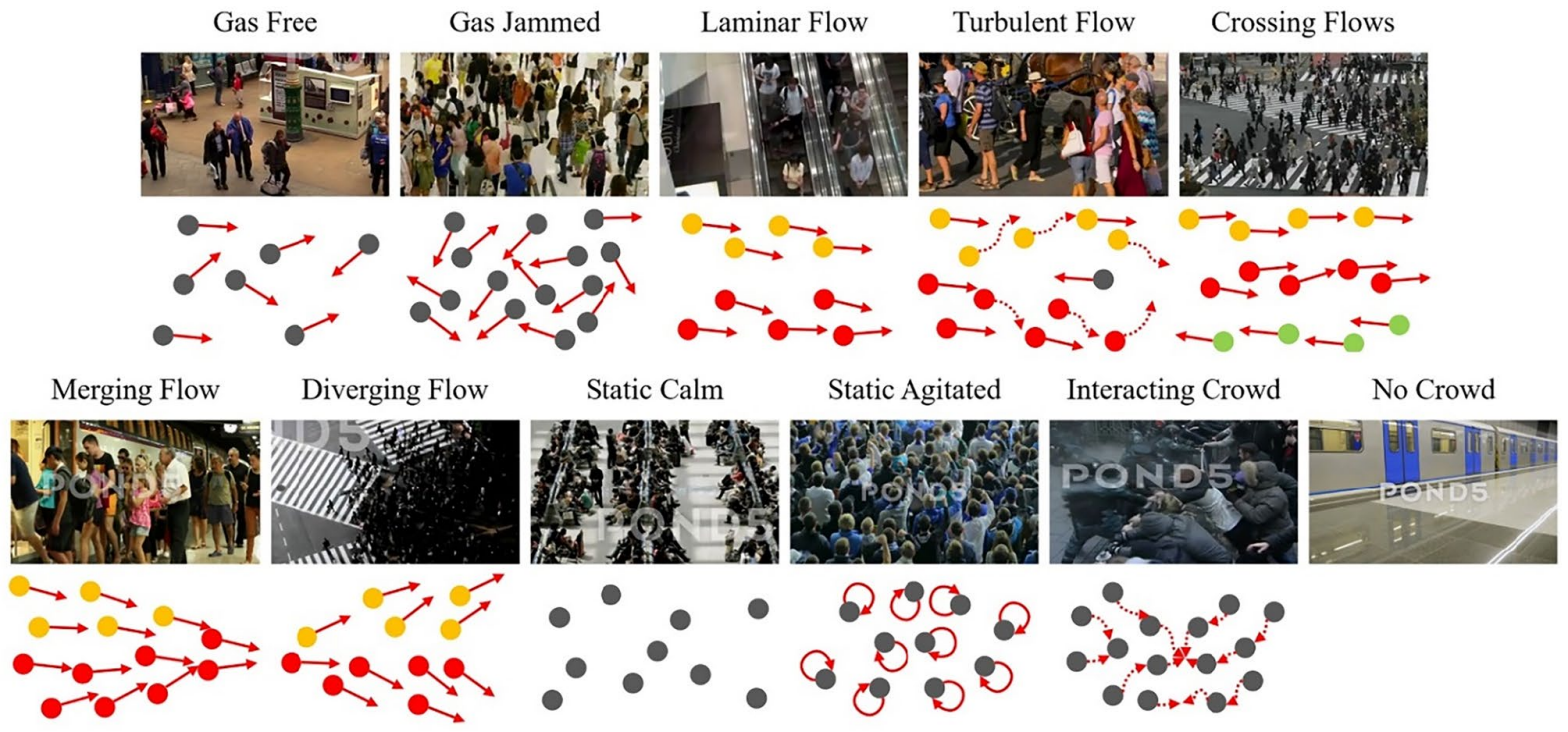
Crowd behavior of each crowd behavior class in the Crowd-11 dataset. Dots represent crowds, gray represents individuals, and colors represent groups. Arrows also indicate their flow direction.

The Crowd-11 dataset^10^ is a newly proposed dataset that addresses the challenge of classifying crowd behaviors. The dataset includes over 6000 videos of crowd behavior in various real-world locations, such as streets, subways, airports, shopping malls, and crosswalks. The videos were classified into 11 different crowd behavior classes, as illustrated in Fig. 1, based on characteristics, such as crowds of various densities and movements, movements of individuals, and cases where there are no crowds. This dataset is a valuable resource for studying deep learning-based crowd behavior classification models. It can be used to train and test models that can accurately classify crowd behavior in complex and dynamic environments, thereby enabling more effective crowd monitoring and management. Therefore, many crowd behavior classification models have recently been developed using the Crowd-11 dataset^21–23,29,30^. However, because of class imbalances^31^, which reduce the ability to discriminate between classes, such as the presence of seven times more laminar flow videos than diverging flow videos, sufficient consideration is required to develop a crowd behavior classification model.

### Crowd behavior classification model

Most deep-learning-based classification methods that use the Crowd-11 dataset rely on 3D CNNs. In addition to the proposal for the Crowd-11 dataset, convolutional 3D (C3D)^29^, an early 3D CNN model, and V3G^10^, a model that transforms C3D into a two-stream form, were proposed and shown to be effective in classifying crowd behavior in the Crowd-11 dataset. Subsequently, the inflated 3D ConvNet (I3D)^3,21–23,30^, which extended GoogLeNet^32^ to three dimensions and showed good performance in human action classification, was applied to crowd behavior classification using the Crowd-11 dataset, achieving better accuracy. The I3D significantly improves the model accuracy using a two-stream method^33^ that combines the results of learning the RGB and optical flow (OF) frames separately in two networks with the same structure. Thus, the aforementioned disadvantages were compensated by merging spatial features through RGB and temporal features through OF.

However, the aforementioned method has a challenge in that the algorithms for calculating OF require a significant amount of computation^34^. Furthermore, even if the model is used, there is an issue with the accuracy of classification for turbulent, crossing, merging, and diverging flows, which do not have a significant difference in crowd behavior. Hence, there is a growing need for a new model that can infer differences in crowd behavior more accurately and quickly.

### Loss function for crowd behavior classification

Currently, in multiclass classification problems, determining a single class to which specific data belong is the most common method, and various loss functions have been proposed and used for this purpose. Among the most widely used loss functions, methods that utilize classification errors, such as the mean squared error (MSE)^35^ and cross-entropy (CE)^36^, are the most commonly used. In particular, CE is the most commonly used loss function because it can easily streamline the learning process in deep-learning-based classification methods and exhibits constant performance in both binary and categorical cases. However, because CE is intended to assign equal weight to all given classes and optimize them, it faces problems, such as class imbalance, which can occur because of the small amount of data or sparse characteristics in the learning process^37^. In particular, class imbalance^31^ reduces the ability to discriminate between classes with similar characteristics even when the amount of data is small. To solve this issue, various loss functions, such as focal loss^38,39^ have been developed and are continuously being researched. However, there is currently no proposal for a loss function that specializes in crowd behavior classification. Thus, a new loss function needs to be proposed for more accurate crowd behavior classification.

## Three-dimensional atrous inception module (3D-AIM)

**Figure 2:**
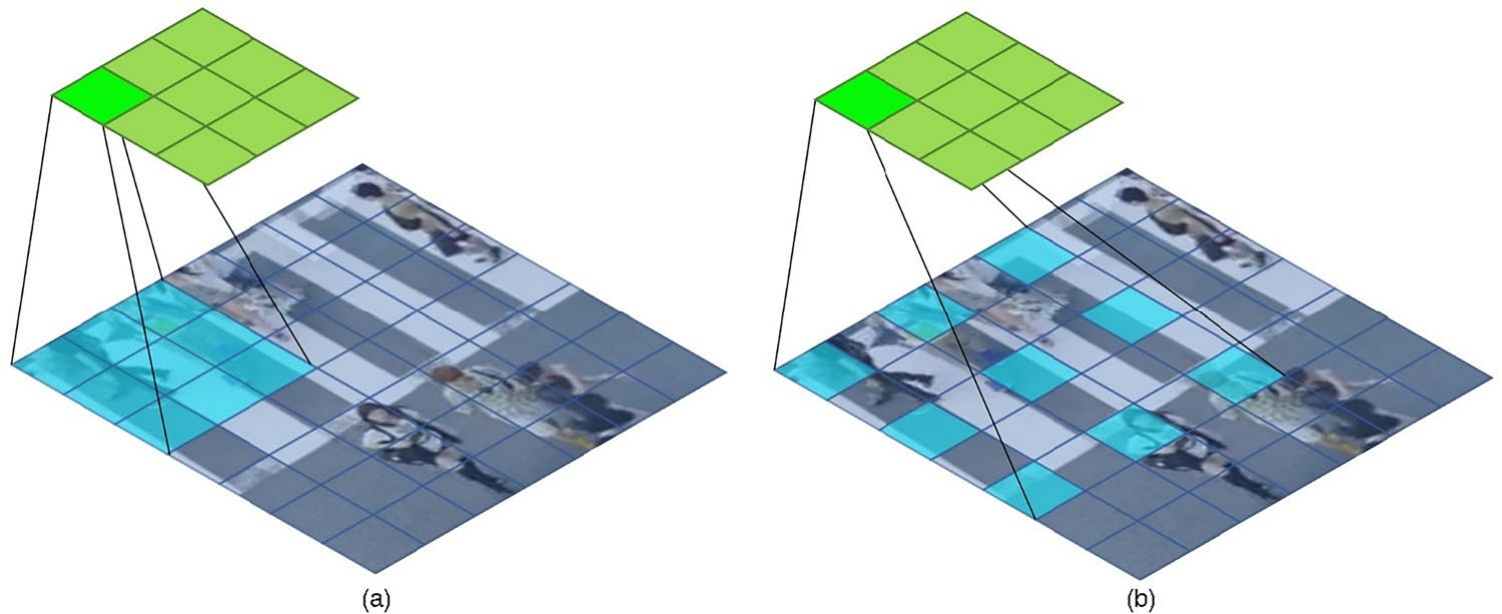
Illustration of the difference in the receptive field between a typical convolution and an atrous convolution for a 3 × 3 kernel.(**a**) typical convolution (**b**) atrous convolution (dilation = 2).

In *3D-AIM*, atrous convolution is used, which can dynamically change the receptive field of the kernel to solve the problem in which the general convolution does not sufficiently consider the interaction between crowds outside the fixed receptive field range, as shown in Fig. 2a. Thus, the interaction between crowds, which is an important factor in crowd behavior classification, can be fully considered within various ranges, enabling a more accurate crowd classification. To describe *3D-AIM*, the following subsection first explains its basic structure *3D-AIM*. Subsequently, the overall network architecture for crowd behavior classification using *3D-AIM* is explained.

### Atrous convolution for crowd behavior classification

The main advantage of atrous convolution, as illustrated in Fig. 2b, is its ability to increase the receptive field while maintaining the same number of parameters by inserting a constant dilation rate between the pixels accepted by the kernel. In a typical convolution, there is a limitation in extracting only spatial information from a limited range of images owing to the fixed-size kernel, that is, fixed receptive field^40^. This limitation results in inadequate spatial information extraction in certain areas, such as object detection and segmentation^24^. Similarly, in crowd behavior classification, a fixed receptive field may lead to the improper extraction of complex interaction features over a wide range.

A method of increasing the kernel size to effectively solve the problem caused by the fixed-size receptive field was proposed, but it led to an increase in the computation cost. Consequently, a new method called atrous convolution, which inflates the receptive field by adding spaces between the kernel elements, has been proposed. This method allows the extraction of spatial information from a wider range at a lower cost^41^.

In this study, we propose *3D-AIM* that extracts and utilizes the characteristics of interactions that can occur in various ranges by simultaneously utilizing atrous convolutions with receptive fields of different sizes. Through this, more accurate crowd behavior classification is possible by more effectively extracting interactions between crowds required for crowd behavior classification.

**Figure 3:**
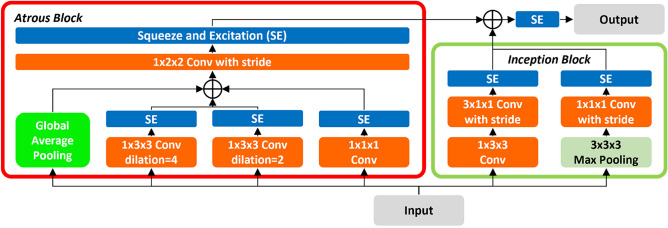
Detailed structure of the* three-dimensinal atrous inception module* (*3D-AIM*).

Figure 3 shows the structure of *3D-AIM*, which extracts the characteristics required for crowd behavior classification based on atrous convolution layers along with squeeze and excitation (SE)^42–44^ to focus on the main channels. It comprises two blocks: an atrous block that extracts spatial features from each video frame, and an inception block that extracts spatial and temporal features from a limited video frame range to extract crowd movements. The atrous block creates two atrous convolutions with different dilation rates: one convolution to reflect the features of the previous layer as it is, and the other to capture global features on the time series input through global average pooling (GAP)^45^ and merge them. By extracting interaction-related features between crowds from various receptive fields and merging them, crowd interactions that occur at various distances can be extracted. In the inception block, spatial and temporal features are extracted through two 3D convolutions, each with a two-dimensional (2D) filter on the spatial dimension and a one-dimensional (1D) temporal connection^46^. Key features can be extracted through max pooling, enabling the recognition of changes, such as crowd movement occurring in a specific area. This approach is similar to the inception module used in I3D^3^, which has been proven to be effective in capturing complex spatiotemporal features in video data. Thus, the proposed *3D-AIM* enables the extraction of various features related to the crowd, and the model using them enables more accurate crowd behavior classification. This is because *3D-AIM* allows for the consideration of interactions between crowds over a wide range as well as changes in movement in both spatial and temporal aspects. Consequently, a more diverse set of features can be extracted to perform crowd behavior classification using videos, leading to more accurate results.

### Network architecture using 3D-AIM

**Figure 4:**
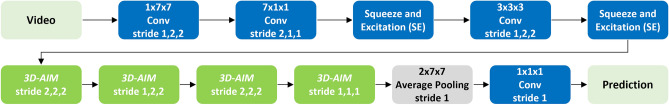
Overall structure of crowd behavior classification network using *3D-AIM*s.

Figure 4 shows the crowd behavior classification network using the proposed* 3D-AIM*s. In our proposed model, we used two convolutions, each with a 2D filter on the spatial dimension and a 1D temporal connection, to explore the spatial and temporal features simultaneously while reducing the resolution and number of frames in the input video. In addition, we applied an attention mechanism using SE. Subsequently, convolution and SE operations were performed to reduce the resolution and number of frames across the entire space–time domain. This approach effectively decreased the computational load while preserving the spatiotemporal characteristics of the input video. Next, we repeatedly used four *3D-AIM*s with varying strides to extract crowd behavior characteristics from the input video. Finally, we performed crowd behavior classification by converting the extracted features into a suitable form for multilabel classification^47^. This enabled us to make individual predictions for each class using the average pooling and convolution layers.

## Separation loss

In this section, we describe the theoretical background of *separation loss*, which is a new loss function that can further improve the accuracy of crowd behavior classification.

### Solving the easy/hard example problem

From the focal loss^38,39^, CE tends to ignore classes that are difficult to predict and have a small amount of data, because a small loss is obtained when many classes can be easily and accurately determined. In particular, CE problems in the crowd behavior classification model using the Crowd-11 dataset are concentrated on classes, such as laminar flow, static calm, interacting crowd, and no crowd with unique features or a large amount of data (i.e., an easy example). Conversely, CE makes it challenging to distinguish between merging or diverging flows with similar features and a small amount of data (i.e., hard examples). Therefore, to improve the accuracy of crowd behavior classification, it is necessary to assign more weight to hard examples such that we can focus on crowd behavior classes that are difficult to classify and increase accuracy.

Different weights are assigned to easy or hard examples using the focal term of the focal loss in *separation loss*. In the *separation loss*, when the ground truth $y=\left\{0, 1\right\}$ is provided, and the predicted value $p\in \left[0, 1\right]$ is obtained from the model’s prediction, the probability ${p}_{t}$ for each class is calculated as follows:

1 $${p}_{t}=\left\{\begin{array}{c}p \\ 1-p\end{array} \begin{array}{l}\text{if}\ y=1\\ \text{otherwise},\end{array}\right.$$

Subsequently, each class's focal loss value *FL* was calculated similarly to the CE by applying the focal term $\left(1-{p}_{t}\right)$ to the original CE in the following manner using ${p}_{t}$:

2 $$FL({p}_{t})=-(1-{p}_{t})\text{log}({p}_{t})$$

The *FL* obtained in this manner can respond to easy or hard examples by generating a small weight in the accurately predicted class (i.e., an easy example) and a larger weight in the inaccurately predicted class (i.e., a hard example). Subsequently, for the *separation loss*, the *FL* calculated for each class was used to improve the class separation.

### Improving class separation

After calculating the *FL* for each class, *SL*, which is the final *separation loss* value, was calculated using *FL*s.

In this step, using the *FL* obtained for each class, the separation weight was calculated to maximize class separation^48^ between the target class and the remaining classes. The separation weight was calculated by adding the focal term of the target class to the average focal terms of the remaining classes. Therefore, it makes it possible to assign a small weight to correct predictions and a larger weight to incorrect predictions. This weight can improve the performance of the model by making it possible to focus on problems that are difficult to classify.

Separation weight *SW* is calculated and utilized as follows for a given video clip $x$ with $n$ classes, where ${p}_{i,t}$ represents the ${p}_{t}$ value calculated for the *i*-th class:

3 $$SW(x)= \sum_{i=1}^{n}\left({ y}_{i}\left(1-{p}_{i,t}\right)+\frac{{(1-y}_{i})\left(1-{p}_{i,t}\right)}{n-1}\right)$$

The final *separation loss* value *SL* using *FL* and *SW* is then calculated as follows:

4 $$SL(x)= SW(x)*\sum_{i=1}^{n}FL\left({p}_{i,t}\right)$$

Thus, because our *separation loss* function uses both focal terms and separation weights, a more accurate prediction for each class and a more accurate classification of crowd behavior classes with similar features is possible.

## Performance evaluation

In this section, we evaluate the accuracy of the proposed network in classifying crowd behavior through various comparative experiments. To ensure an objective performance evaluation of the *3D-AIM* network, we selected two well-established models as baselines: I3D, commonly used in previous studies^21,22^, and X3D^6^, known for human action recognition in videos. We conducted comparative evaluations with these models and our proposed network in the same environment. The following sections discuss the dataset, test models, experimental environment, and the obtained results.

### Dataset and experimental environment

To train and test our models using the Crowd-11 dataset, we used 5987 video clips comprising 64 frames or more from a total of 6146 video clips, excluding videos that were lost online. Because the dataset was not pre-annotated into separate sets for training and testing, we randomly divided the entire dataset into an 8:2 ratio for training and testing.

We used the original I3D and X3D models as baselines for our experiments. For predictions, we utilized 64 frames, both RGB and OF, obtained from the same video. In the case of X3D, we chose the X3D-M model, the medium-sized model within the X3D family. Due to the original model architecture's requirements, we down-sampled the 64 frames to 16 for X3D. We also considered that the performance of the OF-based classification models was significantly influenced by the OF algorithm used. Therefore, we opted to use the traditional TV-L1 algorithm^49^ in all cases. To maintain identical structures, we preserved the original model architecture and modified the number of classes in the last classification layer to 11, which corresponds to the number of classes in the Crowd-11 dataset. Additionally, we implemented both I3D and X3D models using either RGB or OF frames, and two-stream models that combines the results from both modalities. These models were implemented following the same structure as in previous studies using PyTorch 1.10 and Python 3.7. After implementing the models, a comparative evaluation was conducted to assess their performances. For the proposed *3D-AIM* network, we used the model shown in Fig. 4 and implemented it similarly. The parameters and other details of the models are listed in Table 1.

**Table 1:** Details of the models.

Model	#Params	Activation Function	#Input frames	Training
I3D	12.3 M	ReLU^50^	64	Scratch
X3D	3.0 M	ReLU^50^ and Swish^51^	16	Scratch
*3D-AIM*	12.3 M	Mish^52^	64	Scratch

Furthermore, during the training phase, we resized the 64 frames extracted from the video clips to 256 × 256 and then cropped them to 224 × 224 pixels using random crops and random flips to augment the data and prevent overfitting. In the testing phase, we resized the frames to 256 × 256 and then cropped them to 224 × 224 pixels using the center crop to obtain the best possible representation of the input frames. All the models were trained for 200 epochs with a batch size of 16 using two Titan RTX GPUs. We utilized the SGD optimizer with a momentum of 0.9, a base learning rate of 0.1, and a cosine-annealing scheduler to optimize the training phase.

### Experimental result

In this subsection, we present the results of three experiments that evaluated the performance of *3D-AIM* and *separation loss* in crowd behavior classification based on the Crowd-11 dataset. First, we conducted an experiment analyzing the classification accuracy and the confusion matrix to understand its impact on crowd behavior classification performance and to gain further insights into classification errors, such as which classes are frequently confused. Second, we demonstrate that the proposed *separation loss* improves class separation by comparing the t-SNE^53^ results obtained using each loss function. Third, we compared the loss curve of each loss function obtained during the training and testing phases.

In the first experiment, we applied SoftMax^54^, CE, and the proposed *separation loss* to I3D, X3D and *3D-AIM* models to evaluate the effect of *separation loss* on improving the accuracy of crowd behavior classification. In the case of CE, we evaluated the models using binary CE to enable evaluation in a multi-label classification situation, a similar manner to the *separation loss*. Finally, for models using binary CE and *separation loss*, we selected the class with the highest score as the final prediction and compared its accuracy using this approach.

Table 2 lists the accuracy of crowd behavior classification achieved by the I3D, X3D and *3D-AIM* models using each loss function. The results confirmed that loss functions performing multilabel classification, such as CE or *separation loss*, outperformed the commonly used SoftMax in multiclass classification, leading to more accurate predictions for crowd behavior classification. This is because it is possible to learn the unique characteristics of each crowd behavior class individually through multi-label classification, leading to more precise predictions. Moreover, when comparing binary CE and *separation loss* based on multilabel classification, it was found that *separation loss*, which utilizes the focal term and separation weight to address the class imbalance issue and maximize class separation, achieved higher accuracy in crowd behavior classification.

**Table 2:** Crowd behavior classification accuracy according to loss functions.

Input	Model	SoftMax	Binary CE	*Separation loss*
RGB	I3D	71.45%	74.37%	74.54%
	X3D	74.12%	74.37%	75.63%
	*3D-AIM*	76.29%	79.55%	**80.55%**
OF	I3D	73.21%	74.54%	75.63%
	X3D	75.38%	75.88%	77.05%
	*3D-AIM*	76.38%	77.46%	**78.96%**
Two-Stream	I3D	78.63%	80.05%	80.05%
	X3D	80.30%	80.38%	81.72%
	*3D-AIM*	81.72%	82.30%	**83.06%**

Significant values are given in bold.

**Figure 5:**
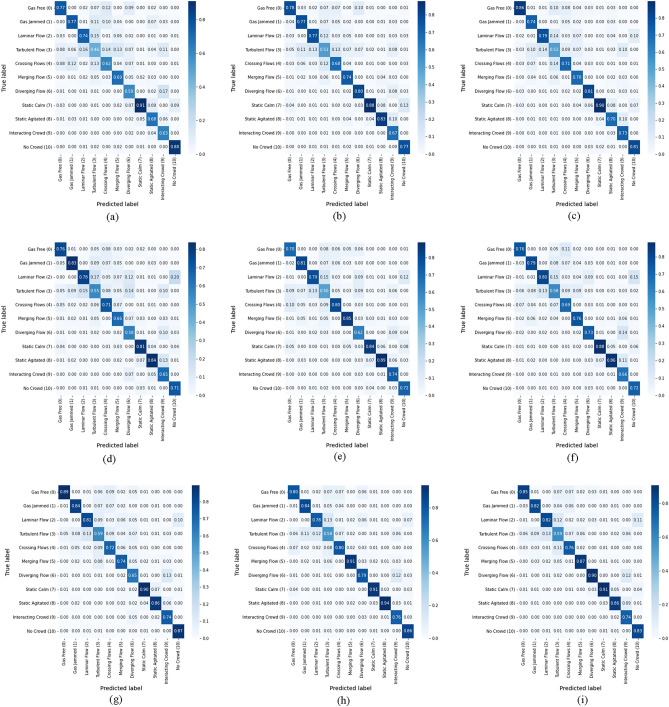
Confusion matrix on the Crowd-11 dataset using I3D with varying loss functions. (**a**) RGB with SoftMax. (**b**) RGB with binary CE. (**c**) RGB with *separation loss*. (**d**) OF with SoftMax. (**e**) OF with binary CE. (**f**) OF with *separation loss*. (**g**) Two-stream with SoftMax. (**h**) Two-stream with binary CE. (**i**) Two-stream with *separation loss*.

**Figure 6:**
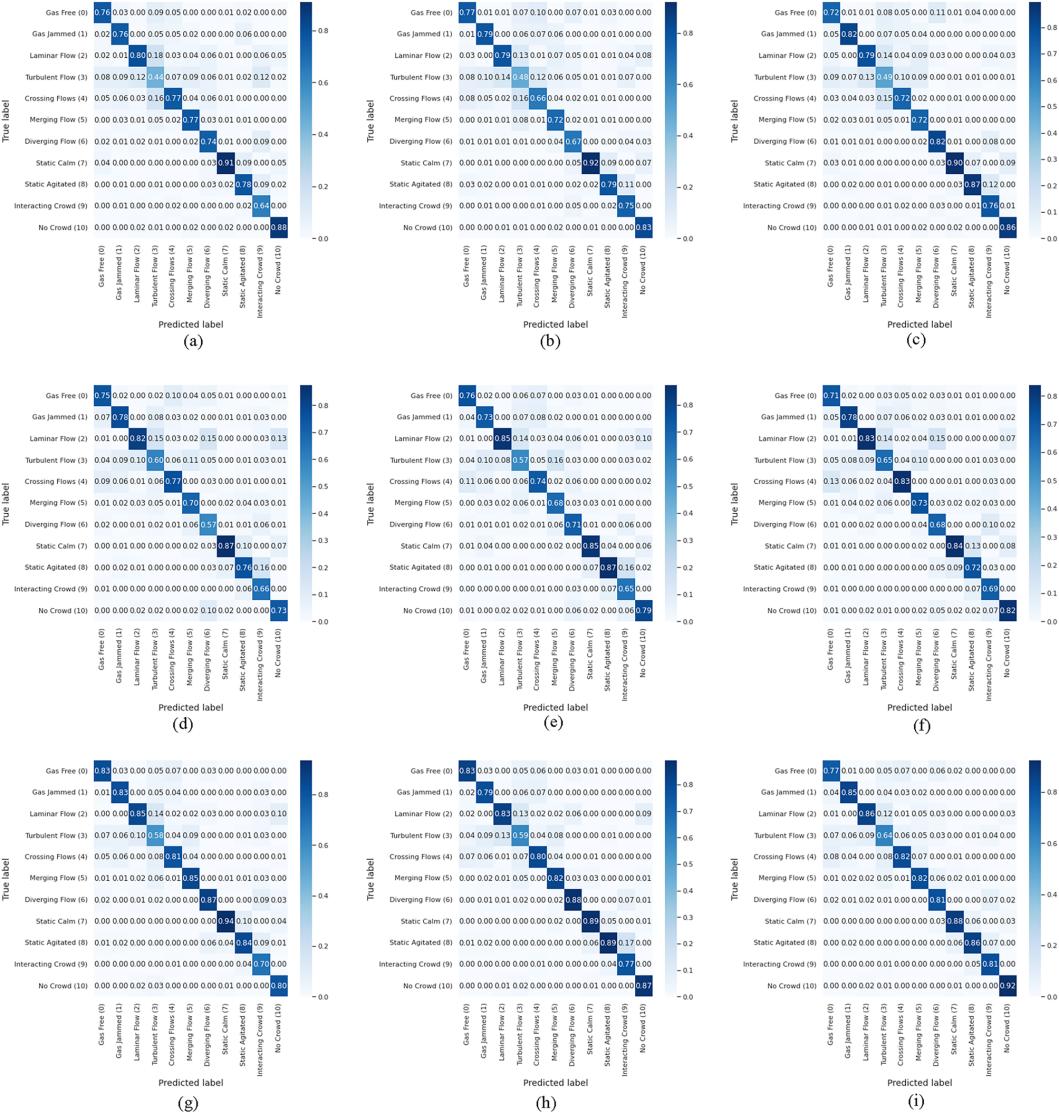
Confusion matrix on the Crowd-11 dataset using X3D with varying loss functions. (**a**) RGB with SoftMax. (**b**) RGB with binary CE. (**c**) RGB with *separation loss.* (**d**) OF with SoftMax. (**e**) OF with binary CE. (**f**) OF with *separation loss*. (**g**) Two-stream with SoftMax. (**h**) Two-stream with binary CE. (**i**) Two-stream with *separation loss*.

**Figure 7:**
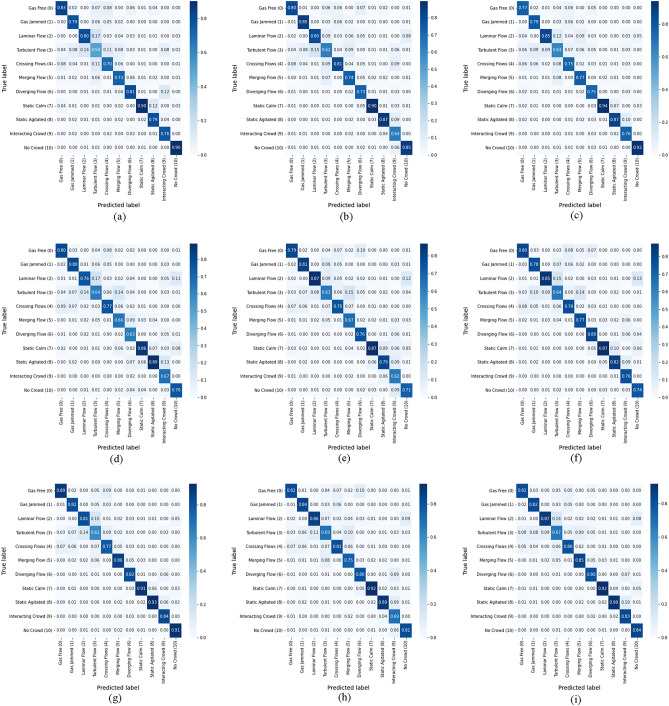
Confusion matrix on the Crowd-11 dataset using *3D-AIM* with varying loss functions. (**a**) RGB with SoftMax. (**b**) RGB with binary CE. (**c**) RGB with *separation loss*. (**d**) OF with SoftMax. (**e**) OF with binary CE. (**f**) OF with *separation loss*. (**g**) Two-stream with SoftMax. (**h**) Two-stream with binary CE. (**i**) Two-stream with *separation loss*.

Figures 5, 6, 7 show the results of the confusion matrix visualization, which facilitates a detailed analysis of the class-specific prediction performance of the models based on each loss function. The confusion matrices visually depict the distribution of the models' predictions compared to the actual crowd behavior classes. Since the number of videos belonging to each class varies, the corresponding counts are also represented as ratios. Key takeaways from these experiments include the superior performance of binary CE and *separation loss* in classifying classes with similar characteristics, such as turbulent, crossing, merging, and diverging flows. SoftMax struggles with these categories due to the inherent difficulty in distinguishing between them. These findings highlight the advantages of employing loss functions beyond SoftMax. Notably, using *separation loss* leads to more refined predictions for turbulent, crossing, merging, and diverging flows, which are characterized by similar crowd behaviors or limited data. Furthermore, these experiments facilitate a more precise comparison of the models' classification accuracy. For example, we can compare I3D, X3D, and *3D-AIM* using the same dataset and loss function. As demonstrated in Table 2, *3D-AIM* consistently outperforms the other models, achieving higher classification accuracy.

**Figure 8:**
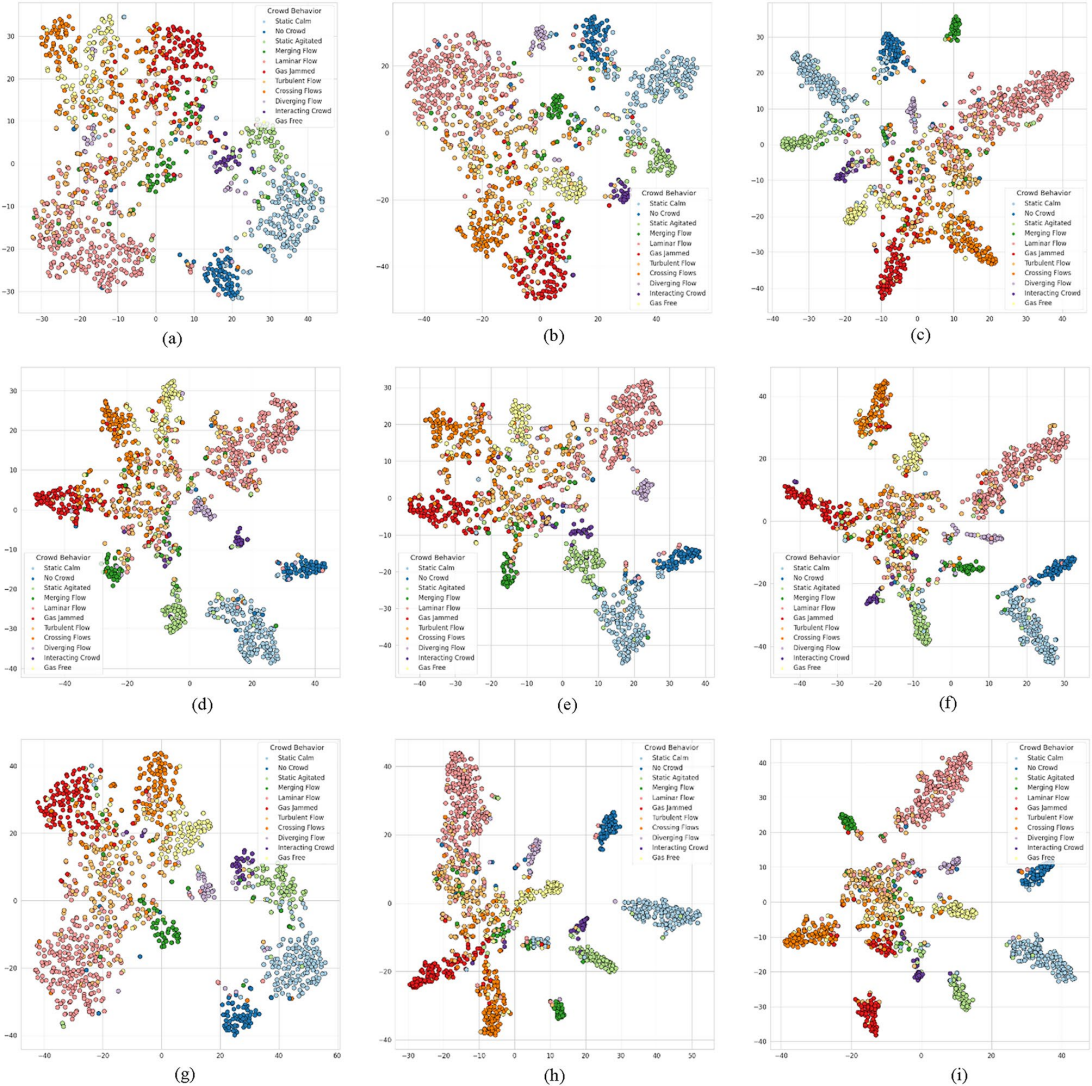
Visualizes the t-SNE embedding of features obtained during the testing phase using RGB with different loss functions. (**a**) I3D with RGB and SoftMax. (**b**) I3D with RGB and binary CE. (**c**) I3D with RGB and *separation loss*. (**d**) X3D with RGB and SoftMax. (**e**) X3D with RGB and binary CE. (**f**) X3D with RGB and *separation loss*. (**g**) *3D-AIM* with RGB and SoftMax. (**h**) *3D-AIM* with RGB and binary CE. (**i**) *3D-AIM* with RGB and *separation loss*.

**Figure 9:**
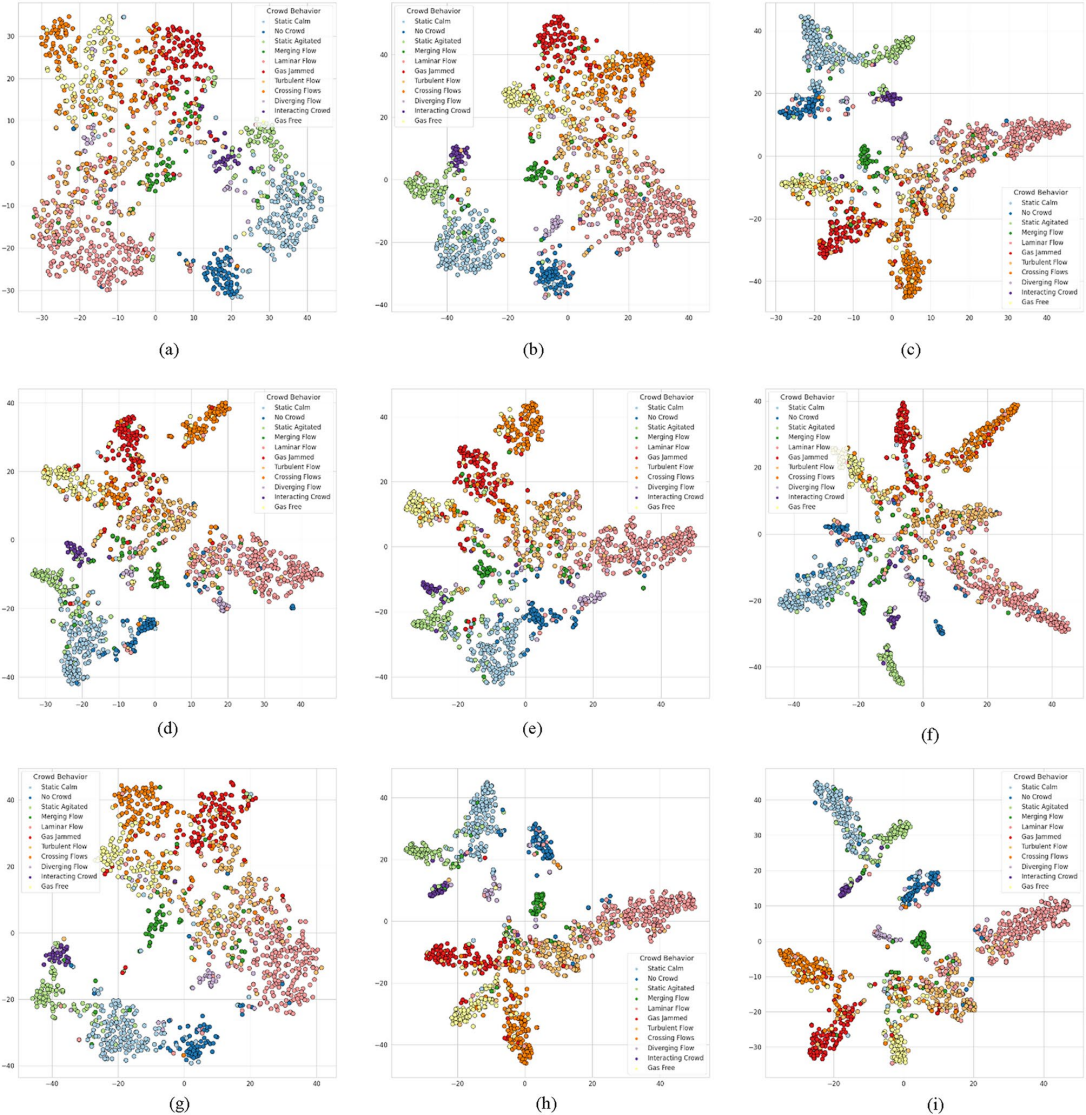
Visualizes the t-SNE embedding of features obtained during the testing phase using OF with different loss functions. (**a**) I3D with OF and SoftMax. (**b**) I3D with OF and binary CE. (**c**) I3D with OF and *separation loss*. (**d**) X3D with OF and SoftMax. (**e**) X3D with OF and binary CE. (**f**) X3D with OF and *separation loss*. (**g**) *3D-AIM* with OF and SoftMax. (**h**) *3D-AIM* with OF and binary CE. (**i**) *3D-AIM* with OF and *separation loss*.

In the second experiment, we demonstrate that the proposed *separation loss* improves class separation by comparing the t-SNE visualization results obtained using different loss functions. This visualization embeds the features into a 2D space, allowing us to inspect the class separations for each loss function. Since the two-stream approach performs the prediction by merging the results of the RGB and OF, only the t-SNE visualization of the RGB and OF features are shown in Figs. 8 and 9. As can be observed from the visualized experimental results, other loss functions are more clearly classifying the class-specific prediction compared to SoftMax. In particular, it can be confirmed that the *separation loss* is performing a more distinct class separation.

**Figure 10:**
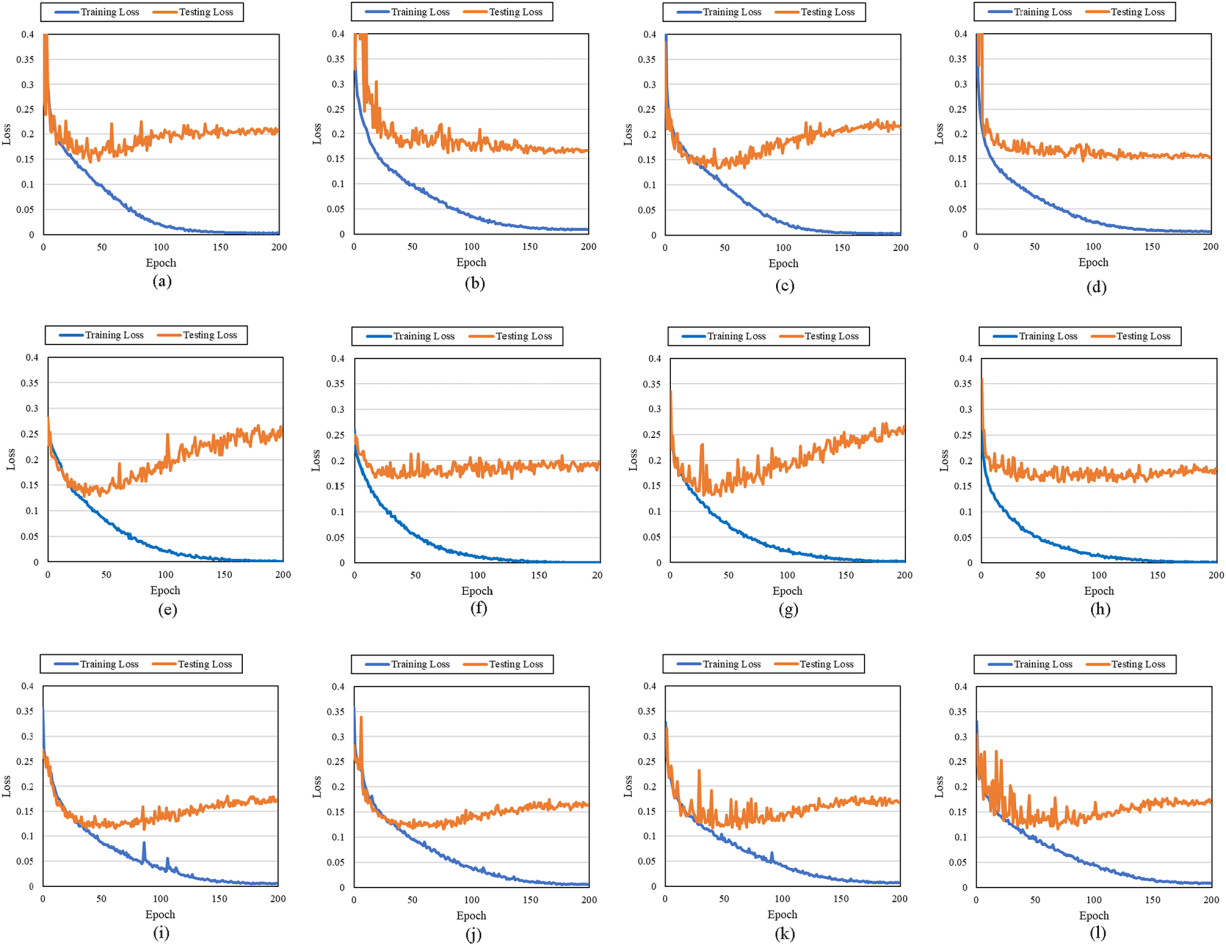
Visualizes the loss curve of binary CE and *separation loss* obtained during the training and testing phase. (**a**) I3D with RGB and binary CE. (**b**) I3D with RGB and *separation loss*. (**c**) I3D with OF and binary CE. (**d**) I3D with OF and *separation loss*. (**e**) X3D with RGB and binary CE. (**f**) X3D with RGB and *separation loss*. (**g**) X3D with OF and binary CE. (**h**) X3D with OF and *separation loss*. (**i**) *3D-AIM* with RGB and binary CE. (**j**) *3D-AIM* with RGB and *separation loss*. (**k**) *3D-AIM* with OF and binary CE. (**l**) *3D-AIM* with OF and *separation loss*.

In the last experiment, we demonstrate the loss curve of the binary CE and *separation loss* obtained during the training and testing phases listed in Table 2. Figure 10 shows that each loss function was used during the training and testing phases, and the loss values were calculated using the same binary CE to ensure a fair comparison. Because the two-stream performs the prediction by merging the results of the RGB and OF, only the loss curves of the RGB and OF are shown. The results show that *separation loss* is more resistant to overfitting than binary CE, and the average loss obtained during the testing phase is also smaller.

These findings suggest that *separation loss* is a more suitable loss function for crowd behavior classification than traditional loss functions, such as SoftMax and binary CE, owing to its ability to effectively address the class imbalance issue and maximize class separation.

## Conclusion

In this study, we propose a novel 3D CNN model, *3D-AIM*, and a new loss function, *separation loss*, to improve the accuracy of crowd behavior classification. Previous studies using typical convolutions had limitations in capturing important features of crowd scenes beyond their limited receptive fields, such as interactions between crowds. To address this issue, we leverage the atrous convolution in *3D-AIM* to increase the receptive fields without increasing the parameters, enabling the extraction of interactions between crowds over a wider range. The proposed *3D-AIM* comprised two blocks: an atrous block and the inception block. The atrous block utilizes atrous convolution to extract spatial features from each video frame, whereas the inception block extracts both spatial and temporal features from a limited range of video frames, enabling the extraction of crowd movements. Furthermore, we introduce *separation loss* to address the class imbalance in traditional classification problems that use SoftMax or CE by focusing more on the key interactions necessary to determine crowd behavior from various and similar interactions between crowds. In *separation loss*, it was possible to improve the accuracy of crowd behavior classification by solving the class imbalance problem using the focal term and increasing the class separation through the separation weight.

To demonstrate the superiority of the proposed method, various experiments were conducted using the Crowd-11 dataset, a real-world dataset for crowd behavior classification tasks. The results show that *3D-AIM* with *separation loss* can more effectively explore the interactions between crowds than existing methods, such as I3D with binary CE, leading to more accurate crowd behavior classification. These results demonstrate that the proposed method can be applied to various applications that require crowd analysis, including crowd management, surveillance, and public safety. Moreover, our approach can be extended to other domains, such as sports and entertainment to analyze crowd behavior and interactions. However, since the *3D-AIM* model was developed using only the well-refined Crowd-11 dataset, it is necessary to apply it to a real video surveillance system and conduct empirical experiments. Therefore, in future work, we plan to validate and improve the performance of the model in real-world environments by implementing and operating *3D-AIM* within an actual video surveillance system.

## Data Availability

The Crowd-11 dataset supporting the findings of this study is available from Dupont et al.^10^ upon reasonable request and with permission. Additionally, all datasets generated and/or analyzed during this study are available from the corresponding authors upon reasonable request.
